# Supplementary material for: Alternative RNA splicing in the endothelium mediated in part by Rbfox2 regulates the arterial response to low flow
Source: eLife. 2018 Jan 2;7:e29494. doi: 10.7554/eLife.29494 (PMC5771670; doi:10.7554/eLife.29494)
Supplement: Supplementary file 1. [file elife-29494-supp1.docx]

**Supplementary file 1: Usage of Source Data**

Figure 1.

B. [Figure 1 - source data 1]

C. [Figure 1 - source data 2; Figure 4 – source data 1]

D. [in GEO, GSE101826]

Supp1 [in GEO, GSE101826]

Supp2 [in GEO, GSE101826]

Supp3 [Figure1 - source data 3]

Supp4 [Figure 1 - source data 2]

Supp5 [Figure 1 - source data 2]

Figure 2.

A. [Figure 2 - source data 1; Figure 4 – source data 1]

B. [Figure 2 - source data 2]

B2, B3. [Figure 2 - source data 1]

Supp1 [in GEO, GSE101826]

Supp2 [Figure 2 - source data 2]

Figure 3.

A. [Figure 3 - source data 1; Figure 4 – source data 1]

E. [Figure 3 - source data 2]

F. [in GEO, GSE101826]

Supp1 [Figure 3 - source data 3]

Supp3 [Figure 3 - source data 4]

Figure 4.

A. [Figure 3 - source data 1; Figure 4 – source data 1]

B. [Figure 3 - source data 1; Figure 4 – source data 1]

D. [Figure 3 - source data 1; Figure 4 – source data 1]

Figure 5.

A. [in GEO, GSE101826]

D. [Figure 5 - source data 1]
