# Supplementary material for: Alternative RNA splicing in the endothelium mediated in part by Rbfox2 regulates the arterial response to low flow
Source: eLife. 2018 Jan 2;7:e29494. doi: 10.7554/eLife.29494 (PMC5771670; doi:10.7554/eLife.29494)
Supplement: Transparent reporting form [file elife-29494-transrepform.pdf]

eLIFE
1st Floor
24 Hills Road
Cambridge CB2 1JP, UK
P 01223 855340
W elifesciences.org
T @elife

eLife's transparent reporting form

We encourage authors to provide detailed information *within their submission* to facilitate the interpretation and replication of experiments. Authors can upload supporting documentation to indicate the use of appropriate reporting guidelines for health-related research (see EQUATOR Network), life science research (see the BioSharing Information Resource), or the ARRIVE guidelines for reporting work involving animal research. Where applicable, authors should refer to any relevant reporting standards documents in this form.

If you have any questions, please consult our Journal Policies and/or contact us: editorial@elifesciences.org.

Sample-size estimation

- You should state whether an appropriate sample size was computed when the study was being designed
- You should state the statistical method of sample size computation and any required assumptions
- If no explicit power analysis was used, you should describe how you decided what sample (replicate) size (number) to use

Please outline where this information can be found within the submission (e.g., sections or figure legends), or explain why this information doesn't apply to your submission:

Detailed definitions of the regulated alternative splicing events (e.g. flow-regulated) can be found in the methods section. No explicit power analysis was done a priori. We hypothesized that, since we would be examining pools of arteries for each condition (e.g. biological replicate 1 of low flow contained 4 arteries, and biological replicate 2 of low flow contained another 4 arteries), biological noise would be dampened and that two or three replicates of each condition would yield reproducible differences.

This proved to be the case, as ~90% of the differences in splicing we found to be significant in vivo between low and high flow arterial intima could be observed in vitro (Figure 1 – figure supplement 4), and were also conserved in the comparison of IgG treated low and high flow arterial intimas (Figure 2).

Replicates

- You should report how often each experiment was performed
- You should include a definition of biological versus technical replication
- The data obtained should be provided and sufficient information should be provided to indicate the number of independent biological and/or technical replicates
- If you encountered any outliers, you should describe how these were handled
- Criteria for exclusion/inclusion of data should be clearly stated
- High-throughput sequence data should be uploaded before submission, with a private link for reviewers provided (these are available from both GEO and ArrayExpress)

Please outline where this information can be found within the submission (e.g., sections or figure legends), or explain why this information doesn't apply to your submission:

eLife Sciences Publications, Ltd is a limited liability non-profit non-stock corporation incorporated in the State of Delaware, USA, with company number 5030732, and is registered in the UK with company number FC030576 and branch number BR015634 at the address 1st Floor, 24 Hills Road, Cambridge CB2 1JP | August 2014

In each figure legend, we state how many biological replicates of each experiment were performed.

We had two cases in which we felt we should exclude data for the cleanest and most accurate analysis.

In the first case, for the data in figure 1, we had three replicates of each condition, however in preparing the arterial flushes for the RNA-seq analysis we had intentionally pooled the cleanest flushes in the first two pools, with the "dirtier" flushes [with some Trizol flowing outside rather than inside the vessel] in the third pool. Upon analysis, we found these first two pools were very similar while the third pool showed variation on some genes – though in general was much closer to the first two pools than any of the other conditions. Given our previously noted concern, we felt it best to confine our analysis to the first two "clean" replicates.

In the second case, we prepared three groups of Rbfox2 KO mice and their controls. The first two groups (replicate 1 of Rbfox2 EC-KO and Rbfox2 control) were similar in some ways to the later replicates, but upon analysis of eGFP to tdTomato ratio (a measure of endothelial purity) we found that replicate 1 of Rbfox2 EC-KO was significantly contaminated (~10%, versus ~1% in the other vessels) by non-endothelial mRNA. Therefore, this was excluded from the analysis shown in Figures 4 & 5.

eLife Sciences Publications, Ltd is a limited liability non-profit non-stock corporation incorporated in the State of Delaware, USA, with company number 5030732, and is registered in the UK with company number FC030576 and branch number BR015634 at the address 1st Floor, 24 Hills Road, Cambridge CB2 1JP | August 2014

Statistical reporting

- Statistical analysis methods should be described and justified
- Raw data should be presented in figures whenever informative to do so (typically when N per group is less than 10)
- For each experiment, you should identify the statistical tests used, exact values of N, definitions of center, methods of multiple test correction, and dispersion and precision measures (e.g., mean, median, SD, SEM, confidence intervals; and, for the major substantive results, a measure of effect size (e.g., Pearson's r, Cohen's d)
- Report exact p-values wherever possible alongside the summary statistics and 95% confidence intervals. These should be reported for all key questions and not only when the p-value is less than 0.05.

Please outline where this information can be found within the submission (e.g., sections or figure legends), or explain why this information doesn't apply to your submission:

Statistical analysis is described in the methods section and in figures when it applies. Bar graphs showing biological replicates show points to reflect the position of the replicates.

Methods for RNA FPKM levels (RSEM), expression (DESeq2) and splicing (MISO) are well accepted, and described in the methods.

Methods for motif enrichment by GSEA test are clearly described in the methods, as we are using the algorithm for a novel application.

Enrichment of gene categories was by GeneGo, using their proprietary methods. These include adjustments for multiple hypothesis testing.

Methods for the analysis of correlation (Pearson) and differences in qPCR (Mann-Whitney) are described in the figure legends, and were computed using GraphPad.

Replicate numbers for each experiment are outlined in the legends.

(For large datasets, or papers with a very large number of statistical tests, you may upload a single table file with tests, Ns, etc., with reference to sections in the manuscript.)

Group allocation

- Indicate how samples were allocated into experimental groups (in the case of clinical studies, please specify allocation to treatment method); if randomization was used, please also state if restricted randomization was applied
- Indicate if masking was used during group allocation, data collection and/or data analysis

Please outline where this information can be found within the submission (e.g., sections or figure legends), or explain why this information doesn't apply to your submission:

Groups were pre-defined (e.g. low flow artery, sham artery, contralateral artery).

Additional data files ("source data")

- We encourage you to upload relevant additional data files, such as numerical data that are represented as a graph in a figure, or as a summary table
- Where provided, these should be in the most useful format, and they can be uploaded as "Source data" files linked to a main figure or table
- Include model definition files including the full list of parameters used
- Include code used for data analysis (e.g., R, MatLab)
- Avoid stating that data files are "available upon request"

Please indicate the figures or tables for which source data files have been provided:

Source data for RNA-seq analysis have been uploaded to GEO (GSE101826), and will be released with the publication.
